# SNPs3D: Candidate gene and SNP selection for association studies

**DOI:** 10.1186/1471-2105-7-166

**Published:** 2006-03-22

**Authors:** Peng Yue, Eugene Melamud, John Moult

**Affiliations:** 1 Center for Advanced Research in Biotechnology, University of Maryland Biotechnology Institute, Rockville, MD 20850, USA; 2 Molecular and cellular Biology Program, University of Maryland, College Park, MD 20742, USA

## Abstract

**Background:**

The relationship between disease susceptibility and genetic variation is complex, and many different types of data are relevant. We describe a web resource and database that provides and integrates as much information as possible on disease/gene relationships at the molecular level.

**Description:**

The resource  has three primary modules. One module identifies which genes are candidates for involvement in a specified disease. A second module provides information about the relationships between sets of candidate genes. The third module analyzes the likely impact of non-synonymous SNPs on protein function. Disease/candidate gene relationships and gene-gene relationships are derived from the literature using simple but effective text profiling. SNP/protein function relationships are derived by two methods, one using principles of protein structure and stability, the other based on sequence conservation. Entries for each gene include a number of links to other data, such as expression profiles, pathway context, mouse knockout information and papers. Gene-gene interactions are presented in an interactive graphical interface, providing rapid access to the underlying information, as well as convenient navigation through the network. Use of the resource is illustrated with aspects of the inflammatory response and hypertension.

**Conclusion:**

The combination of SNP impact analysis, a knowledge based network of gene relationships and candidate genes, and access to a wide range of data and literature allow a user to quickly assimilate available information, and so develop models of gene-pathway-disease interaction.

## Background

Much of our present knowledge of the relationship between genotype and disease comes from statistical studies of the correlation between particular genetic variants and the likelihood of a specific disease. Linkage analysis, which tracks the transmission pattern of genetic markers within a pedigree family, has been successful in identifying over one thousand human monogenic disease genes [1]. On the other hand, there has so far been less success with common human diseases, such as hypertension, Alzheimer's, asthma and cancer. Susceptibility to these is affected by multiple genes, as well as environmental factors. The risk from any single genetic variant is low, so that linkage analysis sample sizes are usually too small to provide statistically significant disease/genotype relationships. Association studies, based on analysis of genetic differences, particularly SNPs, between those with and without a disease in a broader population, are more powerful for detecting such low signals. Approximately 10 million human SNPs have so far been identified [2]. Currently, association studies depend on choosing a subset of these which includes those influencing the probability of disease, or that are in linkage disequilibrium with those that do so. A primary purpose of the SNPs3D resource [3] is to provide a means of selecting candidate genes likely to influence disease susceptibility, and to further select the most relevant non-synonymous SNPs within those genes.

Rapid accumulation of new data on human SNPs, knowledge of the complete human genome sequence, and increasing information on biomarcomolecular interactions is opening the way to a more mechanism based understanding of the relationship between genotype and disease. At present, the relevant information is still very incomplete, and is scattered across many databases and thousands of articles. A second primary purpose of the resource is to collect and integrate as much as possible of the molecular level data relevant to the mechanisms that link genetic variation and disease.

To achieve these goals, the resource is organized into three modules. One module generates lists of candidate genes for any specified disease, based on an analysis of the relationship between the disease and genes, as reflected in the literature. The second module provides a interactive graphical gene-gene network, built from literature associations, known protein-protein interactions [4,5], and existing pathways [6,7]. The third module provides information on the relationship between non-synonymous SNPs and protein function.

The identification of candidate genes and construction of gene networks both make use of simple text mining techniques. Concept profiles are constructed for each disease and for each gene. Each concept (a disease or a gene) is represented by an ordered list of words and terms most closely associated with the concept. The set of words and terms is complied from the contents of the approximately 80,000 PubMed abstracts [8] that have been manually associated with one or more human genes in the NCBI Entrez Gene database [9], using natural language processing [10]. Pairs of concepts, such as two genes or a disease and a gene, are linked by the overlap of their keyterm profiles. We call the resulting gene-gene network a KnowledgeNet, since it is derived directly from knowledge in the literature. Only two types of concept, gene and disease, are discussed in this paper. However, the KnowledgeNet can also be used in others ways, for example investigating the relationship between a biological process (e.g. glycolysis) and genes.

A variety of other computational methods are being developed to automatically extract information from the literature. These methods range from simple technologies which process at the word level and require only a limited linguistic context [11] to state-of-art technologies such as natural language processing (NLP) that handle more complex relations across sentences [12]. So far, these methods have not been used extensively in generally available pathway interfaces. A number of groups, including the Ingenuity Pathway database [13] and the Protein Reference Database [14,15], are developing mammalian pathway descriptions by means of manual curation of the literature. Although these databases provide rather precise data, the human-curation process makes development slow. This problem is becoming more serious as the size of the relevant literature increases. Protein interaction networks have also been built automatically [16-19], using probability models to integrate data from high throughput experiments such as yeast-2-hybrid [20,21] and TAP pull-downs [22].

In SNPs3D, the likely functional impact of non-synonymous SNPs is assessed using two previously developed methods [23-25]. One method makes use of protein structure to identify which amino acid substitutions significantly destabilize the folded state. The results show that up to three-quarters of monogenic disease single residue mutants act in that way [24]. The second method identifies deleterious substitutions through analysis of the extent and nature of amino acid conservation at the affected sequence position [25]. Access to details of both analyzes is provided through the web interface. Links to another publicly available non-synonymous SNP analysis tool are also provided [26,27].

A number of other groups have also developed methods for evaluating the molecular effects of non-synonymous (ns) SNPs [28-36]. Some of these methods form the basis of tools and related analysis that are available through web servers. Facilities range from tools to visualize SNPs in their three dimensional context, such as MutDB [26,27,37], TopoSNP [38-40], SAAP [41,42], to detailed analysis of the molecular effects of nsSNPs. For example, SNPeffect [43] provides a comprehensive analysis of nsSNPs at the protein level [33] including stability analysis using FOLD-X [44], and other functional analysis; PolyPhen [45] models SNP effects with both structure and sequence information [30]; SIFT [46] provides sequence analysis of nsSNPs [28].

SNPs3D aims at integrating all of the available data relevant for assessing the likely role of particular genes and SNPs in a disease. The emphasis is on providing the users access to as much of the underlying information as possible, so that they may make informed judgments. To this end, in addition to SNP impact analysis, links are provided to relevant abstracts, the GAD [47,48], OMIM [49,50] and HGMD [1,51] disease databases, GO annotation [52,53], expression profile data [54], and mouse knockout results [55]. Data are updated regularly. Exploration of gene networks and access is to all information is facilitated by a Java based graphical interface.

## Construction and content

### Query interface

Each of the three modules (SNP analysis, gene-gene network, and disease candidate gene lists and networks) is accessed via a separate simple search window, on the site front page.

The candidate gene search window will accept any word or phrase as an entry, and compiles a concept profile, as described below. For SNP analysis and gene-gene networks requests, a hierarchal query string processing procedure is used, providing a wide choice of input name types, including dbSNP IDs, Entrez Gene IDs, RefSEQ IDs, NBCI Gene Symbols, and common protein names, using the following procedure:

1. A query string is first inspected to determine if its composition is consistent with a dbSNP ID, Entrez Gene ID or Refseq ID. If one of these name types is identified, the query is searched against the corresponding list of possibilities, and if a match is found, appropriate results are returned.

2. If the type of ID cannot be identified, the query string is first treated as a NCBI gene symbol, and searched against that set. If an exact match is found, results are returned.

3. If no exact match to a gene symbol is found, the string is searched against all words in the NCBI Gene summaries of each gene. Any hit adds to a list of high ranked possible genes.

4. This hit list is supplemented by a search of the query string against all the PubMed abstracts associated with each gene in the NCBI Gene Database. The number of times the query string is found in the abstracts for a gene provides a ranking weight. Finally, the user is invited to choose the appropriate gene from the ranked list of possibilities.

5. If a search completely fails, the user is offered an alternative search window, with explicit query string categories.

### Literature dataset

The abstracts of all the medline entries associated with each gene in the NCBI Gene database [56] are the source of words and terms. In the current version, there are, 80,249 Medline references linked to 19,228 human genes. Word types are identified using SVMtagger [10]. Keyterms are constructed from single nouns and adjectives, adjective/noun pairs, and continuous strings of words classified as adjectives or nouns. For example, the phrase 'blood pressure' occurring in an abstract would result in three keyterms: 'blood', 'pressure', and 'blood pressure'. Terms occurring only once are removed. There are currently a total of 266,337 keyterms.

The number of occurrences of each keyterm 'KW' in all the abstracts ('Total_count(KW)' is retained, as well as the number of occurrences of each keyterm in the abstracts associated with each gene 'G', 'Count(G, KW)', and the fraction of all occurrences of each keyterm that are associated with each gene is calculated as:

F1(G, KW) = Count(G, KW)/Total_Count(KW)

### Construction of the gene-gene relationship matrix

The interaction strength L(i, j) between every pair of genes i and j is calculated as:

L(i, j) = ∑_KW _F1(G_i_, KW) + ∑_KW _F1(G_i_, KW)

where the sum is over all keyterms common to the two genes, excluding any found in more than 300 genes. More studied genes have more associated abstracts in the NCBI Gene database, so that this expression upweights interactions involving those. Comparison with a more egalitarian gene-gene weighting, based on a dot product sum similar to that used for the disease/gene linkage, suggests that an emphasis on the hub-like genes is useful for including links to relevant but more weakly coupled genes.

Because of memory constraints, the interactions are stored as a sparse matrix, retaining a maximum of 200 interacting genes per gene. A few well studied genes, such as P53, have more than 200 genes linked with significant scores (greater than the mean element value of the sparse matrix). However, in almost all cases, these elements will be included in the list of associations for other genes.

### Generation of a candidate gene list for a disease

Given a disease name, a list of candidate genes is generated as follows:

A. The subset of abstracts relevant to the disease is identified:

1. Any abstract containing the full disease name, for example, 'breast cancer' is selected.

2. If this procedure results in less than 20 abstracts, and the disease name consists of more than one word, a further search of abstracts is made for the combination of words, for example 'breast' AND 'cancer'.

3. If less than a total of ten abstracts are selected, the process is aborted, returning a message of 'Not enough abstracts to build a profile'.

B: A keyterm profile is generated for the disease, using the selected abstracts. All Keyterms are ranked by the fraction of disease abstracts that contain them:

Rank(KW) = Count_abstracts(D, KW)/[Total_abstracts(KW) +50]

where 'Count_abstracts(D, KW)' is the number of abstracts for disease 'D' containing the keyterm 'KW', and 'Total_abstracts(KW)' is the total number of abstracts containing the keyterm. A pseudo count of 50 is added to reduce noise. The top ranking 40 keyterms are selected, providing Rank(KW) is at least 0.1.

C: The overlap of the disease keyterms with those of each gene is calculated:

1. The number of times each selected keyterm 'KW' occurs in the abstracts associated with the disease 'D', 'Count(D, KW)', is determined, and the relative frequency is calculated as :

F2(D, KW) = Count(D, KW)/Total_Count(KW)

2. The strength of association of the disease 'D' with a gene 'G' is calculated as the dot product of the relative frequencies of the disease keyterms with the relative frequencies of those same keyterms in that gene:

SD(D, G) = ∑_KW _F1(G, KW).F2(D, KW)

where the sum is only over the up to 40 keywords selected as the keyterm set of disease 'D'. The association strength is deliberately biased towards the keyterms most strongly associated with the disease, as opposed to associated with particular genes.

D: Finally, all genes with a non-zero score are returned as candidates.

### Database setup

**Figure 1:**
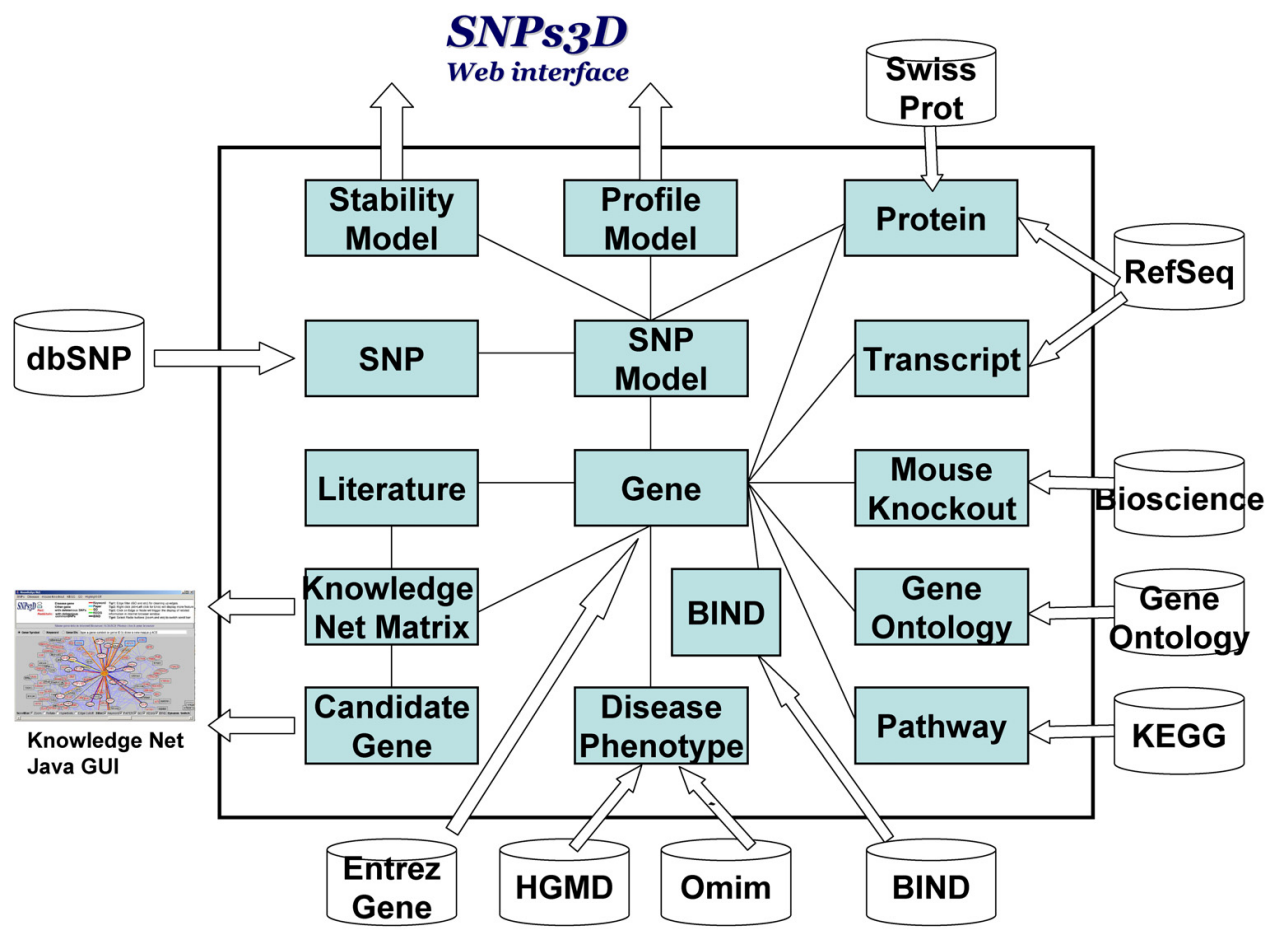
**Database Schema. **The Blue blocks represent individual modules, which may be single or multiple MySql tables.

The database is implemented in MySQL [57]. As shown in figure 1, the central table is 'Gene', an up-to-date list of human genes from the NCBI Entrez Gene database. The Gene table is linked to other master tables: The SNP model table contains our stability and profile analysis of SNPs. There is a table of keyterms for each gene, and a table of PubMed abstract IDs for each gene. The KnowledgeNet matrix table contains the pairwise gene-gene interaction strengths, and there is also a disease/candidate gene matrix. Some other tables linked to the Gene table are: the Transcript table (RefSeq mRNAs); the Protein table (RefSeq proteins); the phenotype and disease-tables (NCBI OMIM and human gene mutation database (HGMD)); Mouse knockout table (Bioscience mouse knockout); pathway (KEGG), protein-protein interactions (BIND); and protein function (GO).

### Web interface

SNPs3D is served using Apache software running on a Linux PC and with web pages derived from an early open source version of PHP-NUKE [58].

### KnowledgeNet graphical interface

The interactive graphical interface for displaying gene-gene relationships is based on open source Java code [59]. Genes form nodes in a graph and gene-gene relationships are edges. Clicking links and symbols leads to more detailed information. Symbol shape; font style; symbol, edge and font color as well as hover-over windows are used to provide as much information as possible. Gene symbol shape conveys whether or not that gene is involved in disease, gene symbol text color indicates whether there are deleterious SNPs. Subsets of genes containing one or more SNPs with population frequencies above some threshold may be highlighted (identifying those most likely to be involved in complex traits). A maximum of 300 genes are displayed in the graphical interface. These are genes most strongly associated with a query gene or a query disease. The threshold for displaying links between genes is adjustable to show only those most strongly linked, or all possible connections. Links may also be based on KEGG pathway connections or direct protein-protein interaction information, extracted from BIND [4]. Left clicking on a gene provides immediate access to all the gene specific information, including SNP analysis using the stability [24] and profile methods [25] and the NCBI Gene summary, as well as pathways, dbSNP entries and homologs.

Content for the graphical display can be generated using the list of genes associated with a reference gene or a disease (the candidate genes, with the strongest linked gene as initial center), or a specified list of genes. All gene lists may be edited. One important feature is the ability to redraw the graph, using a selected node as the new center, allowing the user to smoothly navigate through adjacent regions of the knowledgeNet matrix. A pull down menu provides a list of all displayed genes, and any gene may be highlighted in the network via this list. Right clicking on a node provides facilities for highlighting genes which share certain properties with the reference gene, such as KEGG pathway, associated papers, or sequence homology. Left clicking in a gene brings up its SNP analysis.

## Utility

### Analysis of SNPs in each Human gene

A primary function of the SNPs3D resource is to provide a way of identifying those non-synonymous SNPs that are likely to have a deleterious impact on molecular function *in vivo*, so these may be included in association studies. An analysis of the likely functional impact of all human non-synonymous single base variants in the HGMD (as of 02/09/2002, 9,625 variants in 696 genes) [1] and dbSNP (Build 124, 29,485 SNPs in 11,303 genes) databases [2,60] is provided, using the previously developed methods [24,25]. Links to another available analysis [26,27] are also included. The analysis is organized by gene. The structure/stability method (23[24]) requires knowledge of structure. Availability of experimental structures or sufficiently accurate structure models limits coverage to about 37% of monogenic disease variants in HGMD and 10% of variants in dbSNP. Greater availability of sequence information compared to structure allows a much higher fraction of variants to be analyzed (92% and 57% HGMD and dbSNP respectively) with the sequence profile method.

Both methods make use of a machine learning technique, the support vector machine (SVM), to assign each SNP as deleterious or non-deleterious to protein function. The SVM is trained on monogenic disease data, so that the definition of deleterious is 'sufficiently damaging to protein function *in vivo *as to be consistent with a monogenic disease outcome'. Benchmarking has yielded false positive and false negative rates of 15% and 26% for the stability method and 10% and 20% for the sequence profile method. The higher false negative rate for the stability method reflects the fact that only stability effects on *in vivo *function are included. Approximately 30% of the non-synonymous SNPs in dbSNP are assigned as deleterious. Very few of the dbSNP cases are known to be associated with monogenic disease, and so most the deleterious ones are candidates for contributing to complex disease traits. As illustrated later, in many cases, low impact on the phenotype is likely the result of network level buffering against loss of function for individual proteins.

Details of the analysis of each SNP are provided on additional pages. For the profile model, a user can inspect the multiple protein sequence alignment from which the result is derived. For the structure/stability model, feature values (for example, surface accessibility, electrostatic interactions and hydrophobicity) are provided, as well as an interactive molecular graphics interface (powered by Jmol, [61]) displaying the affected residue in its three dimensional structural context.

### An example of deleterious SNP analysis

**Figure 2:**
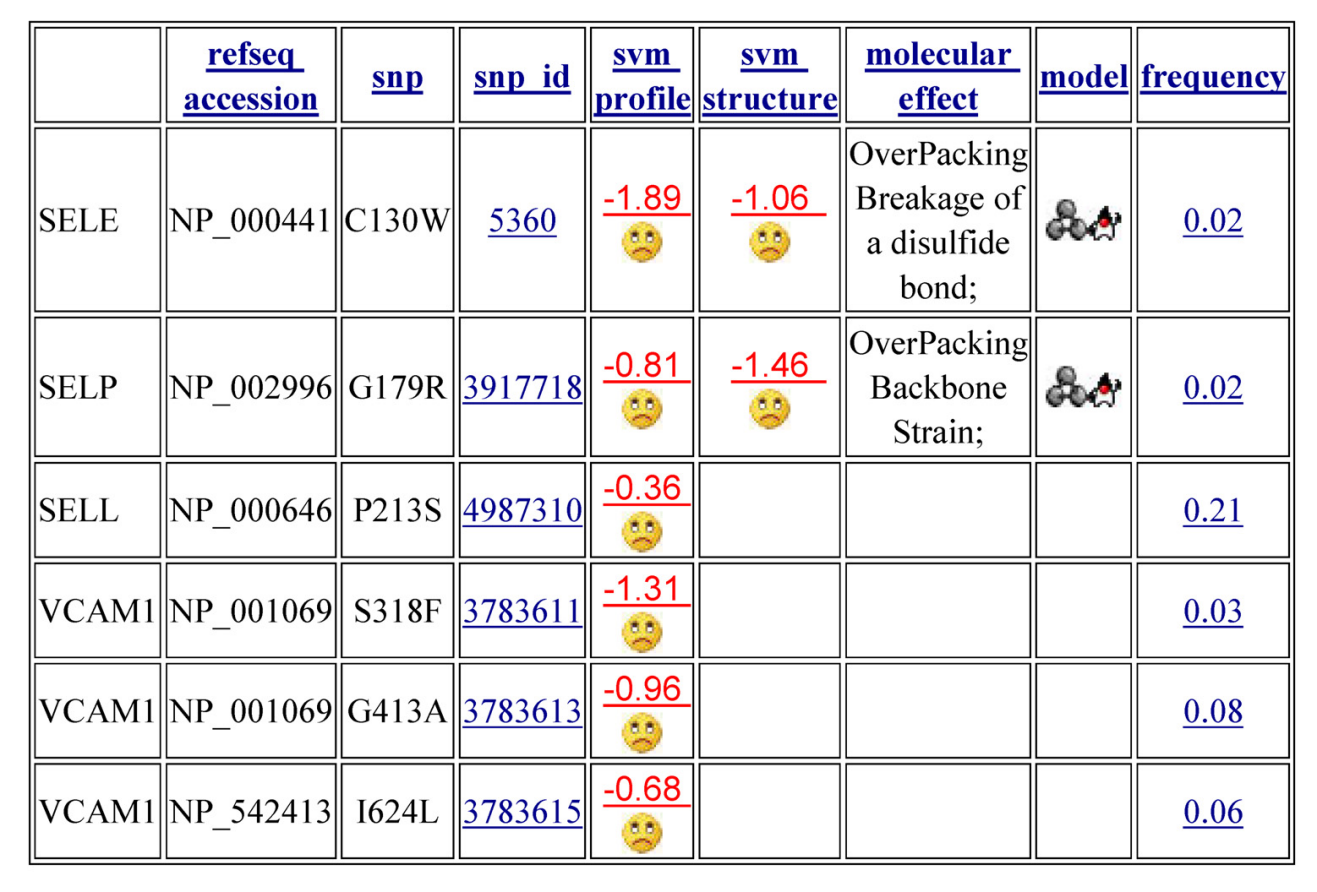
**Example interface page of candidate SNPs for inflammation related disease. **Two support vector machine (SVM) models, based on sequence profiles [25] and structural stability [24] are used to analyze SNPs in candidate genes for inflammation. SNPs are classified as deleterious (negative SVM score) or not to protein function in vivo. SNP population frequency information is extracted from the NCBI dbSNP database.

To illustrate the SNP analysis process, we consider SNPs in the selectins, proteins involved in the early inflammatory response, playing a role in the accumulation of blood leukocytes at sites of inflammation. SNP analysis for relevant genes may be accessed by typing a disease or process name into the corresponding search window. Entering 'inflammation' returns a ranked list of genes with abstracts containing that term, hyperlinked to their SNP analysis pages. Entering a more specific search term, such as 'selectin' returns a list of relevant genes, including the members of the selectin family SELE, SELP and SELL, as well as proteins they interact with. Entering a specific gene name, such as SELE, takes the user directly to the analysis of SNPs in that protein. Figure 2 shows a composite of the screen information for some inflammation related SNPs in selectins E, P and L and VCAM1. Each of these SNPS is classified as deleterious by the sequence profile method (indicated by the negative SVM scores). The SNPs in SELE (C130W) and SELP (G179R) are also analyzed by the structure/stability model, and are found to be deleterious by this criterion as well (a disulfide bridge is broken in SELP, there is overpacking and backbone strain in SELP). As discussed below, further insight into the relationship between these SNPs and the inflammatory response is provided by consideration of the inter-gene relationships.

### Gene-gene relationships

Concept profile overlaps were used to score the relationship between all pairs of human genes in the current NCBI Entrez Gene database. Table 1 shows part of the resulting gene-gene relationship matrix, involving hypertension genes. Angiotensin-converting enzyme (ACE) and angiotensinogen (AGT) share 96 specific keyterms, such as 'sodium intake', 'renin-angiotensin-system' and 'blood pressure'; generating a very strong (43.8) link between them. Many of the shared keywords also have relatively high weights. (That is, the frequency is high in abstracts for these genes, compared with all abstracts). In contrast, the link between ACE and arginine vasopressin (AVP) is much weaker, with a score of 0.8, (still above the average for non-zero relationships in the matrix, which is 0.5). There are only two shared keyterms between these genes: 'polydipsia' and 'hypotension'. 'Hypotension' represents a true concept overlap between these two genes, since both are involved in the regulation of blood pressure. 'Polydipsia' is a symptom found in more than one disease. One of these is Autosomal dominant familial neurohypophyseal diabetes insipidus (ADNDI), some times caused by a missense mutation in AVP [62]. Mutations in ACE have also been shown to be a risk factor in a different disease, schizophrenia, for which polydipsia is also symptom [63]. Thus linkage of ACE and AVP through this term is a not a consequence of their joint role in blood pressure regulation. These indirect linkages are a source of noise in the matrix, but are generally rare.

**Figure 3:**
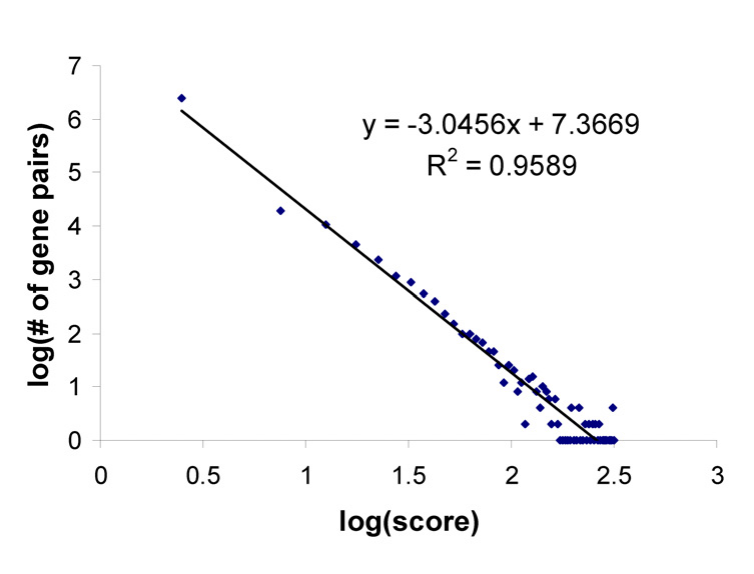
**Log-log plot of linkage scores in the gene-gene KnowledgeNet. **Scores follow an approximately power law distribution, with a few very high scoring relationships (up to a value of 300), and many relatively weak ones.

Figure 3 shows that the distribution of scores between gene pairs has an approximately power law distribution, with many scores near the minimum of 0.001, and a few high scores of up to 300. Pairs of genes which are in the same KEGG pathway [6] tend to have a stronger link than others, with median and mean scores of 0.5 and 2.5, while for all genes the corresponding values of 0.2 and 0.5 respectively. When only those pairs of genes involved in physical interactions included in the BIND database [64] are considered, the median and mean are dramatically higher, at 3.2 and 9.0 respectively. Note that it is not our aim to reproduce either of these known gene-gene relationships, but to introduce a more general, literature based measure.

**Figure 4:**
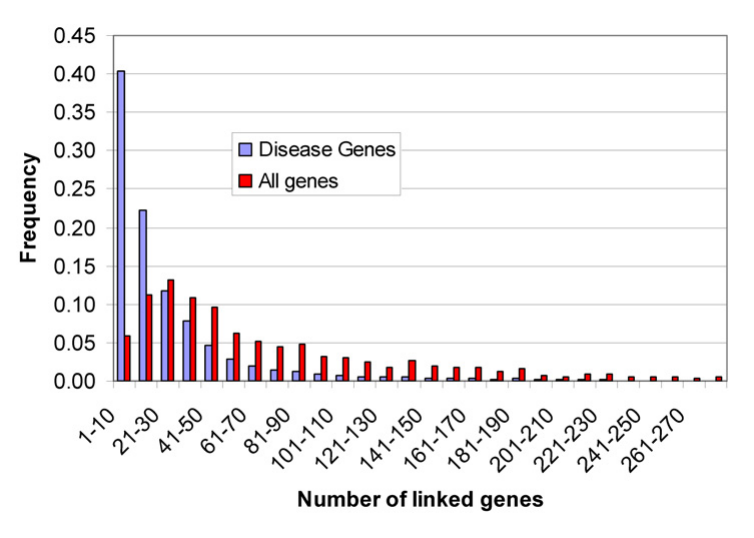
**Distribution of the number of links to each gene in the gene-gene KnowledgeNet. **Blue bars show the distribution for all genes with at least one link (15,799) and red, the distribution for 1669 linked HGMD monogenic disease genes. The tail is truncated – the highest linkage is 493, for TP53. Genes with no interactions above the threshold score of 0.5 are not included.

Figure 4 shows the distributions of the number of gene links, for monogenic disease (defined by inclusion in the HGMD database [1] and all genes. Disease genes tend to be linked to more genes than non-disease genes, reflecting the fact that they are usually well studied, and have been placed in a network context.

### Using the gene-gene KnowledgeNet to investigate SNP-phenotype relationships

The SNPs in figure 2 are classified as significantly deleterious to protein function, and are in genes involved in the inflammatory response. However, none of these SNPs is known to produce a disease phenotype. We next illustrate how the KnowledgeNet can be used to investigate the complex relationships between the effect of these SNPs on protein function and the disease phenotype, through network level buffering against defective protein components. For simplicity, we consider one pair of genes with deleterious SNPs, Selectin E and selectin P. The sidebar on the SNP analysis page provides direct access to a wide range of information relevant to this question, including OMIM, pathways, GO annotation, mouse knockout results, and tissue specific expression data, and relevant abstracts. Clicking 'Gene Graph' in the left sidebar creates a Java window displaying the gene-gene relationships centered on SELE.

A large amount of information is accessible through the Java interface. At the moment, we are specifically interested in possible buffering mechanisms that shield the phenotype from these deleterious SNPs. One such buffering mechanism is overlapping protein function, and many proteins with overlapping function are homologous [65]. Right clicking on the E-selectin node triggers a popup menu, including an option for highlighting all sequence homologs of that node in the graph. L-selectin and P-selectin are seen to be homologous to E-selectin, suggesting possible functional redundancy. The redundancy of selectins E and P is supported by the information obtained from the mouse knockout link in the same menu, which reveals that single mouse knockouts of each gene produce a mild phenotype, while the double knockout is severe [66]. Further support is provided by inspection of the expression profiles for the selectins, which shows a similar tissue specific pattern for Selectin E and selection P, with significant expression in multiple tissues, while selectin L is found in only a few tissues. Thus, an individual homozygous in either one of the deleterious SNPs will likely have a subclinically affected inflammatory response, because of redundancy of function. But an individual with both may have an epistatic interaction between them, and be seriously sick. Both are candidates for inflammation related disease association studies.

### Candidate gene lists for diseases

As discussed in the Introduction, the candidate gene approach is still widely used in association studies. Since knowledge of complex diseases is limited, a comprehensive list of candidate genes and a method of ranking those genes by their disease-relevance is important in designing a good association study. The 'Disease Candidate Genes' module is used to list and rank candidate genes by building a concept profile for the disease and comparing it with the profiles for each human gene. The resulting ranked list of candidate genes can be edited by the user, before further analysis. The Java graphical interface provides access to the resulting gene network, helping a user navigate through the relationships and associated data.

We have pre-complied candidate genes lists for a set 76 diseases, taken from the NCBI on-line book, 'Genes and Disease' [67]. A list for any additional disease may be generated by entering the disease name in the web interface.

**Figure 5:**
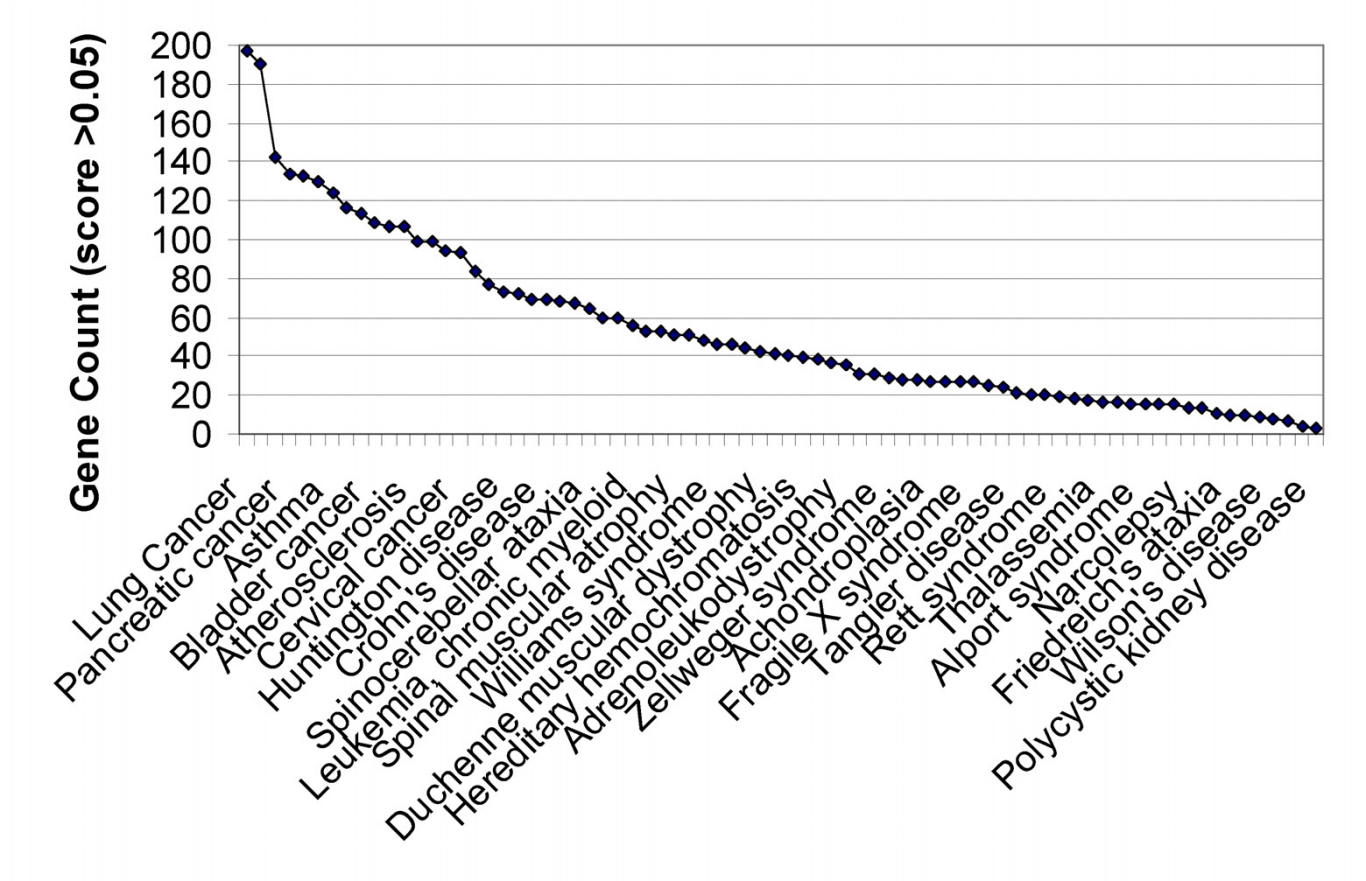
**Distribution of the number of candidate genes for a set of 76 diseases. **The curve shows the distribution using a disease-gene linkage threshold of 0.05. Cancers and common human diseases tend to have many candidate genes, but monogenic diseases typically have more than one candidate as well.

Table 2 lists the 16 diseases associated with the most genes, using an association threshold of 0.05. (Disease-gene profile overlaps have scores ranging from 0 to 24.5 with a mean of 0.04). Figure 5 shows the distribution of the number of genes using this threshold. Cancers tend to have the largest number of candidate genes, with the highest value of 197 genes for lung cancer. Next ranking are well studied common diseases such as asthma, hypertension, inflammation, obesity, Alzheimer's disease, epilepsy, atherosclerosis and deafness. The number of genes associated with a particular disease primarily reflects the complexity of phenotype, but may also partly reflect the current state of knowledge. Not surprisingly, nominally monogenic diseases tend to have the least number of candidate genes. However, these are often not monogenic in this analysis. For example, Phenylketonuria (PKU) has 14 associated genes. As expected, in this case the primary disease gene (PAH – phenylalanine hydroxylase) has a very high linkage to the disease, with a score of 23, while all other genes have scores less than 0.5. The web resource provides a ranked list of candidate genes for each disease.

In all, 2,582 genes are associated with one or more of the 76 pre-complied diseases, using a threshold score of 0.05. TP53 is associated with the most diseases (23). The number of diseases a gene is associated with increases with the number articles associated with that gene.

### KnowledgeNet analysis of candidate genes and SNPs

Once a candidate gene list is available, it is useful to be able to efficiently access the underlying literature, and to generate a list of deleterious SNPs in the genes of most interest. As an example of this process, we consider one of the pre-built disease candidate lists, for hypertension. Clicking on the disease returns a list of the candidate genes, ranked by confidence of disease relevance, based on profile overlap with the disease. Table 3 shows the top part of the list. Highest ranked are well known hypertension-related genes, for example, angiotensinogen (AGT) and angiotensin I converting enzyme (ACE). Each gene in the list is linked directly to local copies of the relevant abstracts, with color highlighting of appropriate words, so that a user may very rapidly assess the evidence for candidate status. There are also links to OMIM [49] and the NIA genetic association database information[47], providing sources of expert information on disease relevance.

**Figure 6:**
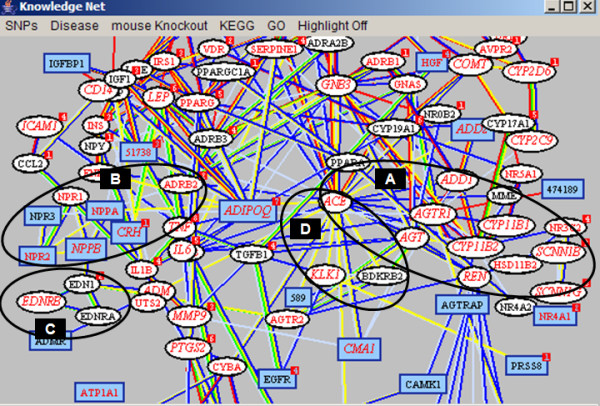
**Graphical Interface for the KnowledgeNet of candidate genes for hypertension. **The four larger ovals circle the clusters of genes in each of the primary blood pressure regulation pathways. Oval symbols are used for genes involved in monogenic disease, rectangular symbols for the rest. Red indicates that one or more population SNPs are classified as harmful at the molecular level. Italic red text indicates that one or more population SNPs with population frequency information are predicted to be deleterious. The length and color of the edges represent the strength of the link between pairs of genes. Red edges link genes sharing the same abstracts. Short edges link genes sharing a large number of biological keywords. Subsets of nodes can be highlighted by a number of criteria, such as membership of the same KEGG pathway, or homology, or SNP frequency.

**Figure 7:**
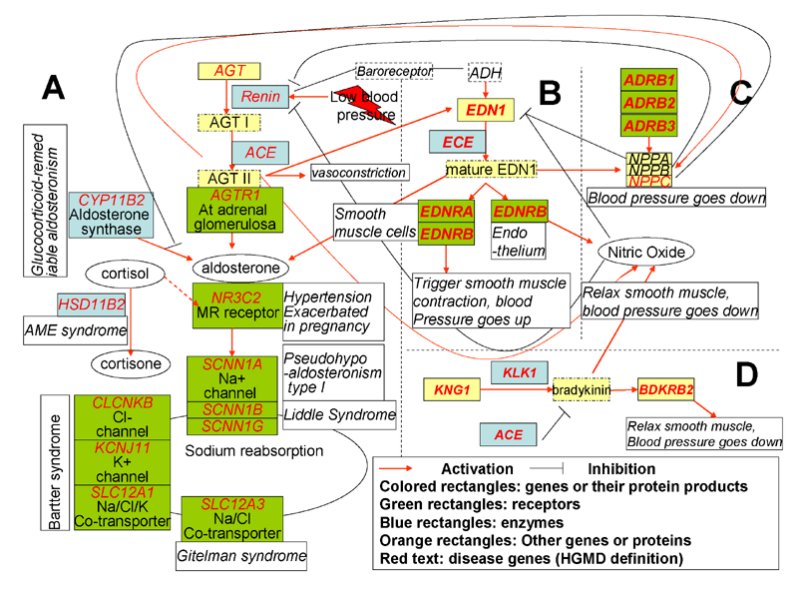
**Simplified view of the four primary candidate pathways involved in hypertension. **A: renin-angiotensin pathway; B: regulation by endothelin (EDN1); C: regulation by natruretic peptide (NPPA, NPPB, NPPC); D: the bradykinin-killikrien pathway.

Since hypertension is a complex trait, with susceptibility related to SNPs in multiple genes as well as the interactions between them, the ability to navigate the network of candidate genes is an important facility of the resource. Viewing the set of candidate genes in the Java graphical interface provides the mechanism for this. Figure 6 shows a screen snapshot of the graphical interface for the hypertension candidate gene network. Strongly associated genes cluster in the display. In particular, in this case, the four primary blood pressure regulation pathways form distinct groups, indicated by the black ovals. Among these, the renin-angiotensin pathway (A), controlling absorption of sodium, is the most studied, and most of its genes have been implicated in monogenic types of hypertension (indicated by the oval gene symbols). The other pathways all influence blood pressure through vascular constriction via: (B), regulation by endothelin (EDN1); (C), regulation of natruretic peptide (NPPA, NPPB, NPPC); and (D), the bradykinin-killikrien pathway. Figure 7 shows a simplified version of the pathways and their inter-relationships, derived from browsing the interface, reviews [68,69], and on-line data [70]. The pathways are highly interconnected. For example, both natruretic peptide and bradykinin also act as antagonists of the rennin-angiotensin pathway, and are able to relax vascular contraction and down-regulate blood pressure. Conversely, ACE, which activates AGT in the renin-angiotensin pathway, can inactivate bradykinin.

This gene/disease network for hypertension provides a number of deleterious SNPs for association studies. A sample of these is shown in Table 4. All are classified as deleterious to protein function by the sequence profile method and the structure/stability method. The first is R333W in rennin, which results in the loss of salt bridge and thus is likely to cause loss of function. Given rennin's role an up-regulator of blood pressure, this SNP is a candidate for involvement in hypotension. The second SNP, I444T, occurs in the hydrophobic core of angiotensin-converting enzyme (ACE) and causes a large loss of buried hydrophobic area. ACE is in the same pathway as rennin, and has an established role in blood pressure related disease. Mutants of ACE have been associated with monogenic-type hypertension [71], and ACE knockout mice show 'subnormal blood pressure, kidney obstruction and widening and thickening of infrarenal arterial vessels' [72]. The third SNP, H66R, is in chymase (CMA1), and changes a key catalytic residue, as well a breaking a salt bridge. The physiological function of chymase is still controversial [73,74]. A SNP upstream of the transcription initiation site of CMA1 has been reported to be associated with hypertensive complications such as HDL cholesterol (possibly related to its lipid metabolism function), but not with blood pressure [75]. The fourth SNP, V193E, in kallikrein (KLK1) results in a buried charge and loss of hydrophobic burial, affecting bradykinin processing.

## Discussion

There are three unique features of the SNPs3D resource. First, it is designed specifically for the analysis of the relationship between SNPs and disease. Second, it constructs gene networks based on conceptual relationships derived from the literature, rather than experimental data. Third, it integrates access to all available and relevant information sources, wherever possible giving the user easy access to the underlying data and literature, so that informed judgments can be made.

We have chosen to construct a network of connections between genes based on how strongly they are coupled in the literature, rather than whether there is extractable information supporting a physical interaction between them. There are two advantages to this approach. First, relevant connections between proteins may be non-physical. For example, genes that are involved in the same complex disease may not directly interact, or even be in the same local pathway, but may never-the-less interact in terms of affecting disease susceptibility. Second, the text mining procedure will capture considerably more information than is currently in any database, or that can be easily formalized in a simple cause and effect pathway description. In this sense, the KnowledgeNet expands on existing pathways descriptions by linking genes with conceptual relationships.

The case studies illustrate how all this works in practice. Analysis of non-synonymous SNPs in the selectins leads to the finding of several that appear to be deleterious to protein function, but which do not directly lead to a disease phenotype. Inspection of homologs in the KnowledgeNet graphical interface suggests a role for functional redundancy in conferring network level robustness, and consulting mouse knockout and expression profile data supports that conclusion. The result also strongly suggests an epistatic relationship between the deleterious SNPs in selectin E and selectin P: An individual homozygous in either one will likely not display clinical symptoms, but an individual homozygous in both will probably have a significantly compromised inflammatory response. In the hypertension example, a list of possible candidate genes is generated. The KnowledgeNet interface allows a user to browse the relationships between those genes, clustering the main pathways, and providing access to analysis of the relevant non-synonymous SNPs. As is often the case, the roles of the some of the genes in disease susceptibility are complicated, and the available information is some times contradictory. For example, for chymase, there is considerable uncertainty of function. Instant access to the relevant literature allows the user to quickly appreciate the subtleties of the current state of knowledge.

We now consider the strengths and weaknesses of the approach in more detail.

Concept profiles for genes are built from the relative frequency of words and terms in PubMed abstracts. In turn, overlap of the profiles are used to identify gene-gene relationships. In practice, the procedure provides intuitively reasonably results, but there is no way of rigorously benchmarking such knowledge generated networks. The method occasionally errs on the side of over-inclusiveness. For example, it is not able to distinguish between statements such as 'protein A is associated with disease B' versus 'protein A is not associated with disease B'. As illustrated in the Results, it is also possible for a disease and gene to be linked by irrelevant factors, such as symptoms common to more than one syndrome. Similarly, gene-gene relationships may sometimes be based on non-pathway related factors. For example the 13 members of the human kallikrein family are tightly coupled, because of many articles that discuss them as a group. In fact, most of the family members operate in quite different pathways. In future, more sophisticated natural language processing technology may be applied to reduce these effects. At present, a concept overlap weighting scheme that emphasizes relationships to 'hub' proteins is used, and ensures that proteins weakly linked to these are included. A weighting scheme that takes into account the number of papers published on a gene may further improve inclusion of relevant weak links. The analysis is limited to abstracts already annotated as relevant to a particular gene. Extension to all pubmed abstracts (currently about 8.5 million) is desirable. In practice, the resource is very effective at narrowing down the amount of literature a user must consult in arriving at an informed position, our main goal.

Concept profile overlaps are also used to provide lists of candidate genes for involvement in susceptibility to particular diseases. There is no gold standard for candidate genes for a disease, with different compilations using different criteria. Comparison of our hypertension list with a hand compiled list for essential hypertension [76], shows informative similarities and differences. That list contains 75 candidate genes rated as 'strong', 57 of which are also in the SNPs3D hypertension set. Nine of the top ten ranking SNPs3D genes are in the hand complied hypertension list. The exception is BMPR2, which is involved in pulmonary hypertension, rather than essential hypertension. The 12^th ^ranking gene in the SNPs3D list, ADRB2, is also not in the hand complied list, but is clearly associated with hypertension in PubMed abstracts. Conversely, some of the additional genes in the hand complied list, such as GALR1, are not linked in any way to hypertension in PubMed, even with a more sophisticated profile based search, and including all abstracts. Their selection may reflect specialized insights on the part of the compliers. Others, such APOC2 and APOC4, are also not associated with hypertension in PubMed, but have a chromosome location covered by a known hypertension marker.

SNPs3D candidate lists can be generated on demand, with little delay, and so have the advantage of taking into account all the current literature. On the other hand, there is a great deal of relevant specialized knowledge in the scientific community that is either not in the literature, or very difficult to extract in a useful way. The Genetic Association Database (GAD) is an archive of human genetic association studies of complex diseases and disorders [47] that provides an alternative approach to compiling the relevant information. Any user may submit information about an association between a disease and a gene, creating a mechanism of capturing community knowledge. We expect that in the long run, the most effective candidate lists will be complied by a hybrid of the two approaches.

SNPs3D analysis is only provided for non-synonymous SNPs. Other sorts of SNPs, particularly those affecting transcription, splicing and perhaps RNA message structure will also play a role in susceptibility to complex trait disease. Little data is available on the relative importance of the different SNP types, although for monogenic disease, the role is relatively small. For example, single base variant effects operating through transcription are quite rare, accounting for 0.5% of cases [1]. Whatever the case, it is clearly desirable to include other classes of SNP. It should shortly be possible to extend coverage in this way, using DNA sequence profiles based on the complete genome sequences of higher eukaryotes.

## Availability and requirements

SNPs3D is freely available at .

## Authors' contributions

PY developed the resource, database and graphical interface. EM, together with PY, developed the profile based text mining. All three authors contributed ideas and concepts. PY and JM wrote the paper.

## Figures and Tables

**Table 1:** Subsection of the KnowledgeNet gene-gene linkage matrix. All three genes are associated with blood pressure regulation. ACE and AGT are strongly linked, other links are near the average value of 0.5.

	ACE	AGT	AVP	...
ACE		43.8	0.8	...
AGT	43.8		0.4	...
AVP	0.8	0.4		...
	...	...	...	

**Table 2:** Diseases with the largest number of significantly associated candidate genes. Cancers tend to have the largest number of candidates, followed by common complex trait diseases.

**Disease**	**Score >0.05**
Lung Cancer	197
Prostate cancer	190
Gastric Cancer	142
Pancreatic Cancer	134
Breast Cancer	133
Diabetes Mellitus	130
Asthma	124
Retinoblastoma	116
hypertension	113
Bladder Cancer	109
Epilepsy	107
Inflammation Related	107
Atherosclerosis	99
Alzheimer Disease	99
Deafness	94
Cervical Cancer	93

**Table 3:** Top ranking candidate genes for 'Hypertension'. The list was complied on the basis of the overlap of the disease concept profile with those of the individual genes. 'Candidate SNPs' shows the number SNPs classified as deleterious in each gene. The 'OMIM' column indicates which genes are associated with essential hypertension in that database. The 'GAD' column shows the number of votes for or against a role for each gene in hypertension in the Genetic Association Database [47].

**Gene Symbol**	**Candidate SNPs**	**OMIM**	**GAD**
AGT	1	Y	N3/Y19
ACE	6		N6/Y24
AGTR1	2	Y	Y11
GNB3	2	Y	N1/Y6
HSD11B2	1		Y1
CYP11B2	2		N1/Y2
BMPR2	0		
ADD1	1	Y	N5/Y4
REN	3		Y3
EDN1	0		

**Table 4:** Example candidate SNPs for hypertension

**RefSNP ID**	**Gene Name**	**Refseq Protein**	**SNP**	**SVM profile**	**SVM structure**	**Structure and Sequence Properties**	**dbSNP ID and Population Frequency**
rs11571098	REN	NP_000528	R33W	-1.63	-0.21	Salt Bridge lost	ss20420843:4% (African American)
rs4976	ACE	NP_690044	I444T	-1.26	-1.15	Hydrophobic Interaction loss	ss6413:5% (Multination) [76]
rs5247	CMA1	NP_001827	H66R	-2.51	-1.49	Salt Bridge lost; key catalytic residue, very conserved	ss6694:10% (Multination) [76]
rs5518	KLK1	NP_002248	V193E	-1.62	-0.70	Buried Charge, hydrophobic interaction decreased	ss6984:5% (Multination) [76]
